# ADAR1 Promotes the Progression and Temozolomide Resistance of Glioma Through p62‐Mediated Selective Autophagy

**DOI:** 10.1111/cns.70168

**Published:** 2025-01-18

**Authors:** Yuyan Zhang, Huiling Guo, Jiahao Bu, Weiwei Wang, Li Wang, Zhibo Liu, Yuning Qiu, Qimeng Wang, Lijuan Zhou, Xianzhi Liu, Liwei Ma, Jianwei Wei

**Affiliations:** ^1^ Department of Neurosurgery The First Affiliated Hospital of Zhengzhou University Zhengzhou Henan China; ^2^ Department of Clinical Laboratory The First Affiliated Hospital of Zhengzhou University Zhengzhou Henan China; ^3^ Key Clinical Laboratory of Henan Province Zhengzhou Henan China; ^4^ Department of Pathology The First Affiliated Hospital of Zhengzhou University Zhengzhou Henan China; ^5^ Electron Microscopy Laboratory of Renal Pathology The First Affiliated Hospital of Zhengzhou University Zhengzhou Henan China

**Keywords:** ADAR1, autophagy, glioma, p62, TMZ resistance

## Abstract

**Background:**

Resistance to temozolomide (TMZ) remains is an important cause of treatment failure in patients with glioblastoma multiforme (GBM). ADAR1, as a member of the ADAR family, plays an important role in cancer progression and chemotherapy resistance. However, the mechanism by which ADAR1 regulates GBM progression and TMZ resistance is still unclear.

**Methods:**

We first constructed stable transfected strains in which ADAR1 was knocked down and overexpressed to investigate the effect of ADAR1 on the first‐line glioma chemotherapy drug TMZ. Subsequently, we validated that ADAR1 induces autophagy activation and used autophagy inhibitors to suppress autophagy, demonstrating that ADAR1 enhances TMZ resistance through autophagy. We further knocked down p62 (SQSTM1) based on the overexpression of ADAR1, and the results showed that ADAR1 regulates selective autophagy through the p62 regulation. Finally, we demonstrated through mutations at both edited and nonedited sites that ADAR1 regulates selective autophagy in an edited dependent way.

**Results:**

Further analysis showed that in the presence of TMZ, elevated ADAR1 promoted TMZ induced autophagy activation. Further research revealed that ADAR1 enhances TMZ resistance through p62‐mediated selective autophagy. Further, ADAR1 regulates selective autophagy in an edited dependent way. Our results indicate a relationship between ADAR1 levels and the response of glioma patients to TMZ treatment.

**Conclusions:**

We found that the expression of ADAR1 is upregulated in GBM and is associated with tumor grade and TMZ resistance. Elevated expression of ADAR1 predicts poor prognosis in GBM patients and promotes tumor growth in vivo or in vitro.

## Graphical abstract

ADAR1 is associated with TMZ resistance. Elevated expression of ADAR1 predicts poor prognosis in GBM patients and promotes tumor growth in vivo or in vitro. Further research found that ADAR1 enhances TMZ resistance through p62‐mediated selective autophagy.

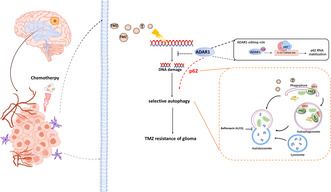

**Abbreviations**
AUC: area under curve
CCK‐8: cell counting kit‐8
CQ: choroquine
DAPI: 4′,6‐diamidino‐2‐phenylindole
DMEM: Dulbecco's modified Eagle's medium
GBM: glioblastoma
GEPIA: gene expression profiling interactive analysis
HRP: horseradish peroxidase
IP: immunoprecipitation
LGG: low‐grade glioma
p62: SQSTM1
RIP‐PCR: RNA immunoprecipitation PCR
ROC: receiver operating characteristics
TMZ: temozolomide
WT: wild‐type

## 1 Introduction

Human glioma is the most common and fatal primary intracranial tumor. It is invasive and malignant and progresses through damage to normal brain tissue, widespread invasion of the entire brain, and resistance to treatment methods [1, 2]. Despite the optimal treatment, the median survival period for patients with glioblastoma (GBM; World Health Organization Class IV) is still 12–15 months [3, 4]. Although TMZ is the first‐line treatment method for GBM patients, drug resistance is inevitable in the vast majority of patients is inevitable [5, 6]. The latest therapeutic progress has involved exploring potential therapeutic methods by targeting the tumor microenvironment of gliomas.

As an oral chemical agent, TMZ has been proven to be effective against various types of tumors, including glioma, metastatic melanoma, lung cancer, colon cancer, and ovarian cancer [7]. Although there is hope for treatment, some patients may experience subsequent tumor recurrence or continue to develop tumors. This represents treatment failure, usually related to drug resistance, which is a major issue in TMZ treatment. Unregulated signaling pathways, DNA repair pathways, self‐defense mechanisms, and persistence of cancer stem cell (CSC) subpopulations are some of the reasons for TMZ resistance [8]. Although the mechanism by which GBM cells develop resistance to anticancer drugs is complex, ADAR1, an important tumor regulatory factor, is currently becoming a key factor in TMZ resistance.

With the deepening understanding of the structure and function of ADAR1, an increasing number of studies are linking it to human disease. Many human diseases, such as autoimmune and autoimmune diseases, cancer, and viral infections, are associated with ADAR1. ADAR1 has been shown to play a role in tumorigenesis, and the development of cancer has been shown to be related to its editorial role, mainly due to its richer expression and unique characteristics [9]. One feature of ADAR1 is that it is located on chromosome 1 arm q, a region that is frequently amplified in cancer [10]. One study revealed that ADAR1 binds to and stabilizes CDK2 through its RNA‐binding domain (RBD), which plays key role in cancer cell cycle progression and therefore promotes the proliferation of glioblastoma in vitro and especially in vivo [11].

Autophagy is a process of degradation through which cytoplasmic macromolecules, such as unnecessary proteins or damaged organelles, are digested by lysosomal enzymes. This process recycles the digested materials into biomolecules, thereby maintaining cellular homeostasis. It involves complex pathways and is regulated by multiple factors. However, nutritional hunger is the most famous autophagy‐inducing factor. Biological stress factors, such as hypoxia or energy expenditure, hormone stimulation, oxidative stress, and drugs, can also increase autophagy in cells [12]. Therefore, autophagy can regulate cell growth, metabolism, and survival and overcome stress states.

Since two pioneering reports introduced the impact of ADAR1 on gastric cancer progression [13, 14], others have reported targets including antizyme inhibitor 1 (AZIN1) [15], the mTOR/p70S6K pathway [16], and phosphatase and actin regulator 4 (PHACTR4) [17]. All these studies consistently demonstrated the oncogenic role of ADAR1 in gastric cancer progression. However, the role of ADAR1 in TMZ resistance in glioma remains unclear. Therefore, investigation of the underlying mechanisms of ADAR1 in TMZ resistance may provide new therapeutic strategies to improve the glioma response to TMZ. We first constructed stable transfected strains in which ADAR1 was knocked down or overexpressed to investigate the effect of ADAR1 on the first‐line glioma chemotherapy drug TMZ. Subsequently, we validated that ADAR1 induces autophagy activation and used autophagy inhibitors to suppress autophagy. Finally, we demonstrated that ADAR1 enhances TMZ resistance through autophagy. We further knocked down p62 based on the overexpression of ADAR1, and the results showed that ADAR1 regulates selective autophagy through the p62 regulation. Finally, we demonstrated through mutations at both edited and nonedited sites that ADAR1 regulates selective autophagy in an edited dependent way.

## 2 Methods

### 2.1 Cell Culture

The human glioma cell lines U251, T98G, and U87 tool cells HEK293FT were obtained from American Type Culture Collection (ATCC). The cells were stored in DMEM supplemented with 10% foetal bovine serum and 1% penicillin/streptomycin. All cells were cultured in a humidified atmosphere at 37°C with 5% CO_2_.

### 2.2 Patient Samples

Patients underwent surgical treatment at the Department of Neurosurgery, the First Affiliated Hospital of Zhengzhou University. Specimens were histologically diagnosed as WHO II, WHO III, or WHO IV (GBM) according to the WHO classification.

### 2.3 Gene Knockdown and Overexpression

ADAR1‐overexpressing and ADAR1‐knockdown lentiviruses were constructed by Genechem Co. Ltd. (Shanghai, China) and transfected into U251 and T98G cells. Cells were selected with 1 μg/mL puromycin (starting from 5 days post infection) and expanded.

### 2.4 Lentivirus Infection and Plasmid

HEK293FT cells (5–6 × 10^6^) incubated in a 10 cm dish for a confluency of 80% were transfected with the following three plasmids using Lipofectamine 3000: 5 μg target plasmid, 3.3 μg pSPAX2, and 1.7 μg pMD2.G Packaging plasmids. After 48 incubations, the cell culture supernatant was filtered using a 0.45 μm filter and concentrated by a Lenti‐X concentrator (Clontech). The lentiviral pellet was resuspended in 2 mL of fresh culture medium containing 4 μg/mL of polybrene and added to 1 × 10^5^ cells cultured in a six‐well plate. Infected cells were then incubated with puromycin or blasticidin S hydrochloride (1 μg/mL) for 48 h post infection for antibiotic selection. The extent of infection of target plasmid was evaluated by western blotting analysis with HA or F‐Flag antibody. Shp62, ADAR (K554E, K555A, K558A, K665E, K666A, K669A, K777E, K778A, K781A) were obtained from OBiO Technology (Shanghai, China) Corp. Ltd. ADAR NM_00111(E912A) were obtained from Genechem (Shanghai, China) Corp. Ltd.

### 2.5 Cell Counting Kit 8 (CCK‐8) Assay

The experimental cells were inoculated into 96‐well plates at 4 × 10^3^ cells per well. Subsequently, the cells were exposed to TMZ, and 10 μL of CCK‐8 solution was added to each well at 24, 48, and 72 h, respectively. The cells were incubated in a 37°C incubator in the dark for 2 h, after which and the absorbance value at 450 nm was measured using an enzyme‐linked immunosorbent assay (ELISA). This experiment was repeated at least three times.

### 2.6 Colony Formation Assay

The transfected U251 cells were inoculated at a density of 500 cells per well into a six‐well plate in triplicate. After 24 h, the cells were exposed to TMZ for 14 days. Then, the cells were washed with PBS, fixed in 4% formaldehyde, and stained them with 0.1% crystal violet at room temperature for 20 min. The number of colonies with at least 50 cells was counted. The colony formation rate was calculated by dividing the number of colonies after 10 days by the number of cells initially inoculated.

### 2.7 Migration Assay

To evaluate in vitro cell motility, monolayer injury tests and Transwell assays were also conducted. For trauma testing, cells are allowed to form a monolayer on the surface of the culture dish, and when they approach 100% cell convergence, a wound is formed by scraping the monolayer with the tip of a pipette. After the cells were scratched, they were cultured in a 5% CO_2_ incubator at 37°C, and images of the wounds were taken at different time points (after the cells were magnified 10 times using a Leica DMi8 microscope). Three independent series of experiments were conducted. The wound area was calculated through the program Image J software (NIH, Bethesda, MD, USA). The percentage of wound closure is estimated by the following formula: wound closure% = [1 − (wound area at Tt/T0)] × 100%. T0 is the time immediately after the trauma, and Tt is the time 24 h after the trauma.

### 2.8 Transwell

A 24‐well Transwell (Corning, NY, USA) chamber system was used to perform cell invasion assays. Then, 2 × 10^4^ cells were added to the upper chamber with 0.2 mL of serum‐free medium. The bottom chamber contained 500 μL of culture medium supplemented with 10% foetal bovine serum (FBS), which was used as a cell attractant. After incubating for 24 h, the chamber was removed, the cells were washed three times with PBS, and the cells were fixed with formalin for 30 min. Then, the cells were washed three times with PBS again, 0.5% crystal violet solution was added for 20 min, and the cells were counted based on digital images of five fields of view randomly captured at 10× magnification. Three independent experiments were conducted. For statistical analysis, we used a bilateral *t* test.

### 2.9 RNA Isolation and qRT–PCR

The total RNA was extracted using TRIzol reagent according to the manufacturer's instructions. The RNA concentration and purity (A260/A280 nm ratio) were evaluated using a NanoDrop ND‐2000 (Thermo Fisher Scientific) system. Total RNA was treated with DNase I, and reverse transcription was performed using the ImProm II reverse transcription system (Promega). Quantitative real‐time polymerase chain reaction (qRT–PCR) was performed using predesigned assays (TaqMan Applied Biosystems Life Technologies) to validate the expression of specific mRNAs. GAPDH served as a control for mRNA normalization. After passing through 2−ΔΔ, the Ct method was used to calculate the relative amount of each matrix. The expression level is expressed as the relative multiple increase compared to the control sample arbitrarily set to 1. All qRT–PCRs were repeated two or three times, at least twice from the independent RT–PCR. The *p* value (bilateral *t* test) was calculated, and the data are presented as mean ± SD. RNA isolated using the SYBR green dye detection system was quantified, and the process was repeated twice. The 2−ΔΔCt method was used to measure the relative expression level of the target. Specifically, in these experiments, the oligonucleotides used were primers. All the reactions were conducted on an Applied Biosystems 7500 rapid real‐time PCR system.

### 2.10 Western Blotting

The proteins were separated from the cells using RIPA lysis buffer. The protein samples identified with a BCA protein concentration determination kit were separated via 10% SDS–PAGE or 12% SDS–PAGE and transferred to polyvinylidene fluoride membranes. After the membrane was blocked with 5% skim milk for 2 h, the PVDF membrane was incubated overnight with antibodies at 4°C. Next, the sections were incubated with goat anti‐rabbit or anti‐mouse IgG bound to horseradish peroxidase (HRP) membranes at 37°C for 2 h, after which the protein bands were visualized via enhanced chemiluminescence.

### 2.11 RNA Immunoprecipitation (RIP)

We used an RNA immunoprecision (RIP) kit to extract RNA for 2 × 107 cells that were lysed and divided into three parts according to 0.8 mL (IP), 0.8 mL (IgG), and 0.1 mL (Input). ADAR1 antibody and an equal amount of IgG antibody were added to the IP and IgG samples, respectively, and incubated for 4° (overnight). Forty microliters of balanced magnetic beads was added in half to IP and IgG samples, incubated at 4° for 1 h, washed thoroughly, and RNA extracted for IP, IgG, and input. Total RNA was treated with DNase I, and reverse transcription was performed using the ImProm II reverse transcription system (Promega). Amplification was performed using 2× SYBR Green qPCR Mix kit with p62 and GAPDH primers and analyzed by SDS‐PAGE.

### 2.12 Immunofluorescence and Confocal Microscopy Analysis

The glioma cells were seeded on poly(L‐lysine) slides and fixed in 4% paraformaldehyde at room temperature for 20 min. The cells were washed twice in PBS, infiltrated with PBS containing 0.1% Triton X‐100 for 10 min, and incubated with PBS containing 0.5% BSA for 1 h. Then, the cells were incubated with antibodies diluted 1:500 at 4°C. The mixture was kept at room temperature for 1 h. The slide was washed and loaded into 50% glycerol in PBS. DAPI was used to stain the nucleus. Confocal imaging was performed on an Olympus Fluoview FV1000 confocal microscope equipped with FV10ASW version 4.1a software.

### 2.13 Nude Mouse Intracranial Model

BALB/c‐A nude mice at 4 weeks of age were purchased from GemPharmatech Co. Ltd. (Jiangsu, PR China). For the intracranial tumor model (randomly divided into four groups), a total of 5 × 10^4^ GBM cells were stereotactically implanted using cranial guide screws. Seven days after the cells were injected, the mice (one group, *n* = 5) were allocated to receive intraperitoneal injections of 30 mg/kg bodyweight TMZ every other day for 28 days. At the end of the experiment, the intracranial tumor model mice were sacrificed. The brains of tumor‐bearing mice were carefully extracted and fixed in 10% formalin and subjected to hematoxylin–eosin (H&E) and immunohistochemical (IHC) staining. All animal studies followed internationally recognized guidelines and national regulations.

### 2.14 Statistical Analysis

The data are expressed as mean ± SD from at least three independent experiments. The statistical analyses we applied in this study included the unpaired *t* test, one‐way ANOVA, and Spearman's correlation. The analyses were performed with SPSS 20.0 (SPSS Inc., Chicago, IL). *p* < 0.05 was considered to indicate statistical significance.

## 3 Results

### 3.1 ADAR1 Is Highly Expressed in Glioma and Promotes TMZ Resistance in Glioma Cells

**FIGURE 1:**
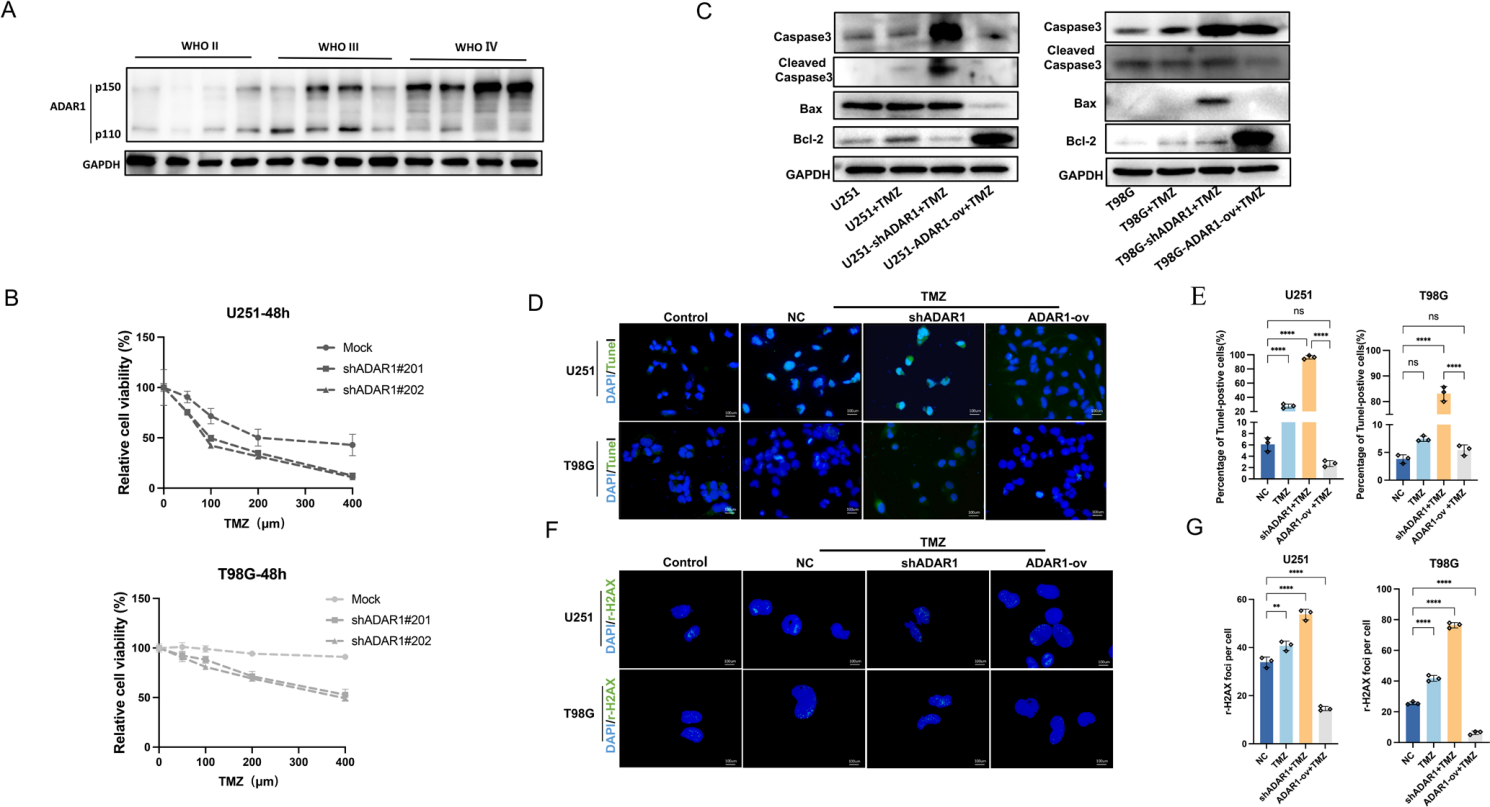
ADAR1 promotes TMZ resistance in GBM. A, Western blot analysis of ADAR1 protein expression in different grade glioma samples. B, CCK‐8 assays in U251 and T98G cells with ADAR1 knockdown treated with 0, 50, 100, 200, and 400 μM TMZ, Statistical significance was determined by two‐way ANOVA with Dunnett's multiple‐comparison test. C, Expression levels of Caspase3, Cleaved Caspase3, Bax, and Bcl‐2 detected by Western blot in U251 and T98G cells with ADAR1 overexpression or knockdown treated with TMZ for 24 h. D, E, IF assays showing TUNEL levels (TUNEL in green and nucleus in blue) of U251and T98G cells with ADAR1 overexpression or knockdown treated with TMZ for 24 h. Scale: 100 μm. The statistic of IF assays are shown in bar plots. Statistical significance was determined by ANOVA. F, G, IF assays showing γ‐H2AX levels (γ‐H2AX in green) of U251and T98G cells with ADAR1 overexpression or knockdown treated with TMZ for 24 h. Scale: 100 μm. The statistics of IF assays are shown in bar plots. Statistical significance was determined by ANOVA. Data are shown as mean ± SEM from three independent experiments. Student's *t* test. **p < 0.01, ****p < 0.0001.

ADAR1 plays an important role in tumors and can regulate the progression of various tumors. Compared to that in normal tissues, ADAR1 was highly expressed in glioma tissues (Figure S1A) using web‐based tools (GEPIA, Gene Expression Profiling Interactive Analysis; http://gepia.cancer‐pku.cn/) [18]. Second, we use the median of ADAR1 expression levels in these 404 patients from their division into high‐risk and low‐risk groups, in the CGGA693 datebase (CGGA, the Chinese Glioma Genome Atlas; http://www.cgga.org.cn/) [19], and the survival curve showed that the high expression of ADAR was associated with poor prognosis in glioma (Figure S1B). These results indicate that the expression of ADAR is negatively correlated with the prognosis of GBM patients. GBMs are aggressive, and pharmacological treatment for GBMs is challenging due to the anatomical characteristics of GBMs (the blood–brain barrier and tumor microenvironment) and increasing resistance to marketed drugs, such as TMZ, the first‐line drug for GBM treatment. Due to physical–chemical properties such as a short half‐life and the increasing resistance in GBM cells, high doses and repeated administrations are necessary, leading to significant adverse events [20]. The correlation between the expression of ADAR1 and the prognosis of GBM patients was verified in the previous results. Compared with gliomas of different grades, higher grade gliomas also exhibited stronger and more significant expression of ADAR1; compared with that in lower grade tissues, ADAR1 in GBM tissues, the protein expression of ADAR1 in GBM was greater (Figure 1A). Therefore, we suspect that the expression of ADAR1 also has a certain impact on TMZ resistance. To analyze the role of ADAR1, we used three different types of ADAR1 shRNAs to knock down endogenous ADAR1 in glioma cell lines (U251, T98G, and U87) through adenoviral infection and overexpressed endogenous ADAR1 in glioma cell lines (U251, T98G and U87) using the same method. Subsequently, we confirmed the transfection efficacy and efficiency through q‐PCR and Western blotting. In the ADAR1 knockdown group, the protein and RNA expression levels were significantly reduced, while in the overexpression group, the opposite was observed (Figure S2A–D). The immunofluorescence results also confirmed the effectiveness of the knockdown and overexpression regimens we selected. Immunofluorescence staining for the cell proliferation marker Ki67 showed that after the same concentration of TMZ was added, the proliferation of shADAR1 cells was significantly lower than that of cells overexpressing ADAR1 (Figure S2E). Through a CCK8 cell proliferation experiment, we found that the knockdown of ADAR1 in glioma cells (U251, T98G) significantly increased the sensitivity to TMZ (Figure 1B). The results for the U251 cell line were consistent with those for the T98G cell line, further indicating that the expression of ADAR1 increased the resistance to TMZ drugs. ADAR1 knockdown enhanced TMZ‐induced apoptosis, as indicated by the induction of Bax, Bcl‐2, and cleaved caspase‐3 in U251, T98G, or U87 cells. However, ADAR1 overexpression inhibited TMZ‐induced apoptosis in these three types of cells (Figure 1C; Figure S1C). We further evaluated TMZ‐induced cell apoptosis using immunofluorescence technology. Moreover, the TUNEL analysis of U251 and T98G cells revealed that when ADAR1 was knocked down, cell apoptosis increased, and ADAR1 overexpression reduced the number of apoptotic U251 and T98G cells (Figure 1D,E). Because ADAR1 inhibits TMZ‐induced cell apoptosis, we speculated that ADAR1 may promote the resistance of glioma cells to TMZ. Immunofluorescence staining of the DNA damage marker r‐H2AX showed that after the addition of the same concentration of TMZ, the extent of DNA damage in shADAR1 cells was significantly greater than that in cells overexpressing ADAR1 (Figure 1F,G), possibly because ADAR1 reduces cell apoptosis by inhibiting DNA damage. These data indicate that ADAR1 plays an important role in mediating the drug resistance mechanism of TMZ in glioma cells.

### 3.2 ADAR1 Promotes the Proliferation and Migration of Glioma Cells

**FIGURE 2:**
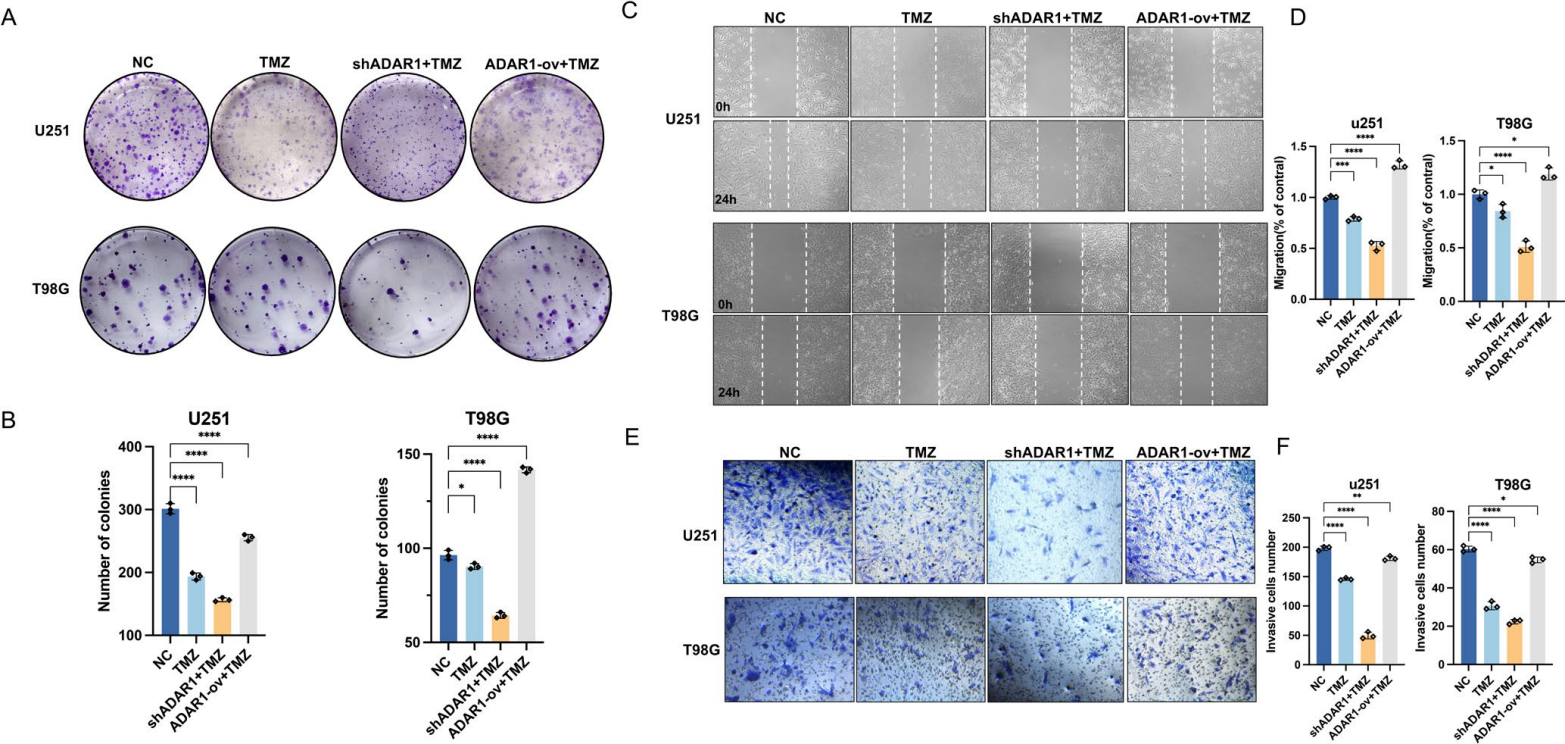
ADAR1 promotes the proliferation and migration of glioma cells. A, B, Colony formation assay was performed to evaluate the viability of U251 and T98G cells transfected with ADAR1 overexpression or knockdown treated with TMZ. Statistical significance was determined by ANOVA. C, D, Wound healing migration assays were performed in U251 and T98G cells transfected with ADAR1 overexpression or knockdown treated with TMZ for 24 h. Statistical significance was determined by ANOVA. E, F, Transwell invasion assays in U251 and T98G cells transfected with ADAR1 overexpression or knockdown treated with TMZ for 24 h. Statistical significance was determined by ANOVA. Data are shown as mean ± SEM from three independent experiments. Student's *t* test. *p < 0.05, **p < 0.01, ***p < 0.001, ****p < 0.0001.

Compared to that in normal brain tissue, ADAR1 mRNA was overexpressed in GBM and LGG (Figure 1A), but only ADAR1 was negatively correlated with patient survival in all three GBM databases [21]. Subsequently, through cloning, we found that, after treatment with TMZ, the proliferation ability of glioma cells significantly decreased after ADAR1 knockdown, which indicated that the resistance to TMZ decreased. After the overexpression of ADAR1, this conclusion also holds true (Figure 2A,B). To evaluate the migration ability of cells in vitro, we subsequently conducted cell monolayer damage experiments and Transwell experiments. The experimental results confirmed that ADAR1 also affects the migration ability of cells in vitro (Figure 2C–F; Figure S3A,B). By analyzing the changes in the area of cell damage and the ability of cells to pass through the upper layer of the small chamber, it is not difficult to see that ADAR1 promotes cell migration and induces drug resistance in glioma cell lines to TMZ. After the overexpression of ADAR1, the ability of cells to migrate in vitro is significantly enhanced.

### 3.3 ADAR1 Promotes TMZ‐Induced Activation of Autophagy

**FIGURE 3:**
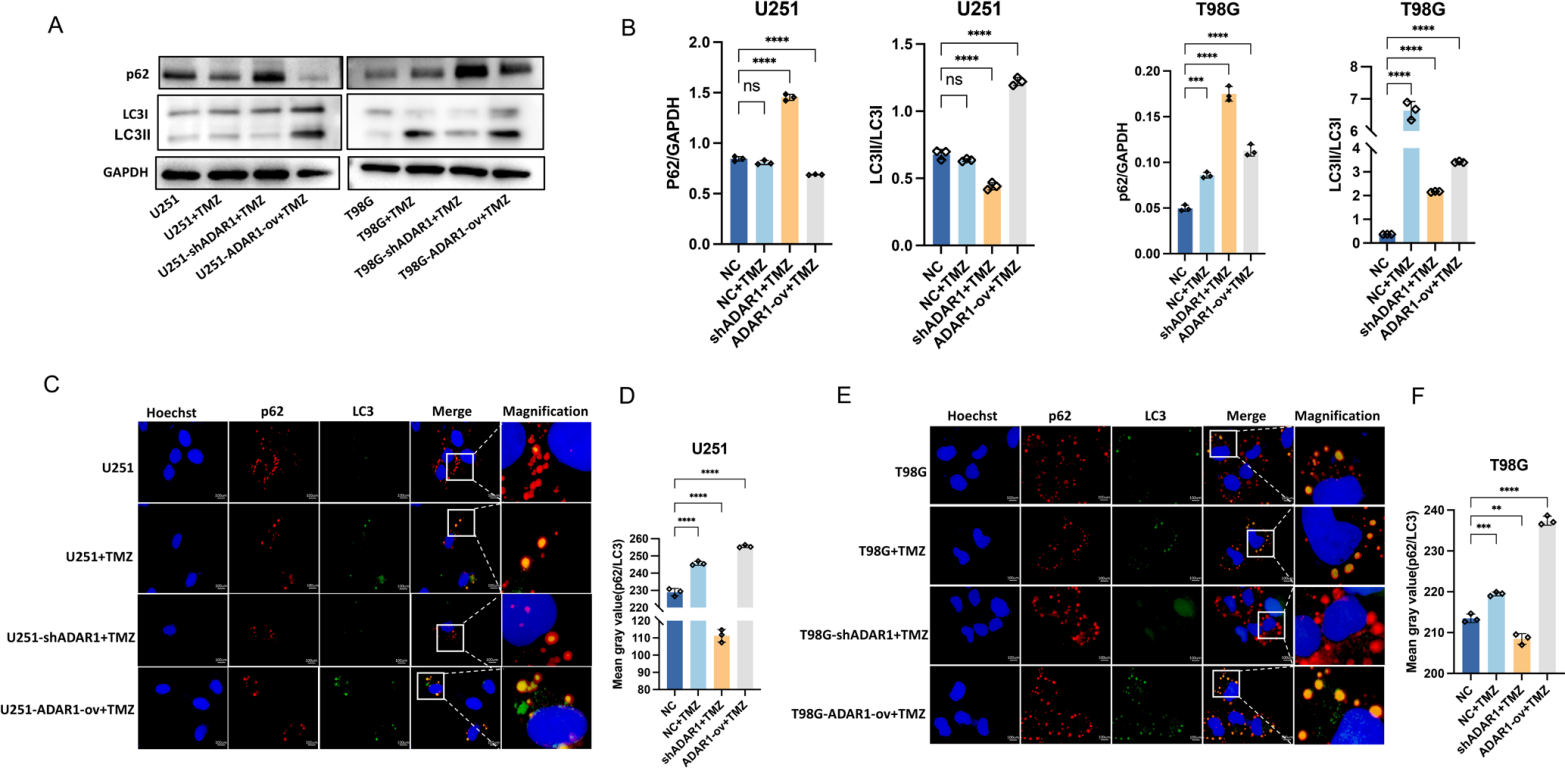
ADAR1 promotes TMZ to induce the activation of autophagy. A, B, Expression levels of p62 and LC3 detected by Western blot in U251 and T98G cells with ADAR1 overexpression or knockdown treated with TMZ for 24 h. Statistical significance was determined by ANOVA. C–F, Immunofluorescence images of p62 and LC3 showed that p62 (red) is co‐localized with LC3 (green). The scale bar in the column represents 100 μm. The statistic of IF assays are shown in bar plots. Statistical significance was determined by ANOVA. Data are shown as the means ± SEMs from three independent experiments. Student's *t* test. **p < 0.01, ***p < 0.001, ****p < 0.0001.

Autophagy plays an important role in tumor progression and drug resistance, and the literature has reported that autophagy is closely correlated with glioma. After phagocytosis, macrophages degrade engulfed cells through LC3‐related phagocytosis, and this blocking effect enhances antitumor immunity [22]. The proteins of the LC3 family (MAP1‐LC3A, B, and C) are structural proteins of autophagosome membranes and are often used as markers of autophagy [23]. p62 is a multifunctional adapter protein involved in the processes of autophagy. In the autophagic degradation of ubiquitinated substrates, p62 first interacts with polyubiquitinated proteins through its ubiquitin‐associated domain [24]. To investigate the effect of ADAR1 on TMZ‐induced autophagy, we combined ADAR1‐knockdowned cells with ADAR1 overexpression and control cells with TMZ in two glioma cell lines and used Western blotting to detect the expression of the LC3 protein and the p62 protein, which are related to autophagy. We found that the expression of the LC3II/LC3I protein was reduced and that the expression of the p62 protein was significantly increased after ADAR1 knockdown. In contrast, after ADAR1 overexpression, the expression of LC3II/LC3I was significantly increased, while the expression of the p62 protein was reduced. These findings indicate that the autophagy pathway is inhibited after ADAR1 knockdown and activated after ADAR1 overexpression (Figure 3A,B; Figure S4A). Immunofluorescence staining revealed the colocalization of two specific markers of autophagy (LC3 and p62) (Figure 3C–F; Figure S4B). In summary, these results indicate that ADAR1 can promote TMZ‐induced activation of the autophagy system. ADAR1 promotes resistance to TMZ through autophagy.

### 3.4 ADAR1 Promotes TMZ Resistance Through Autophagy

**FIGURE 4:**
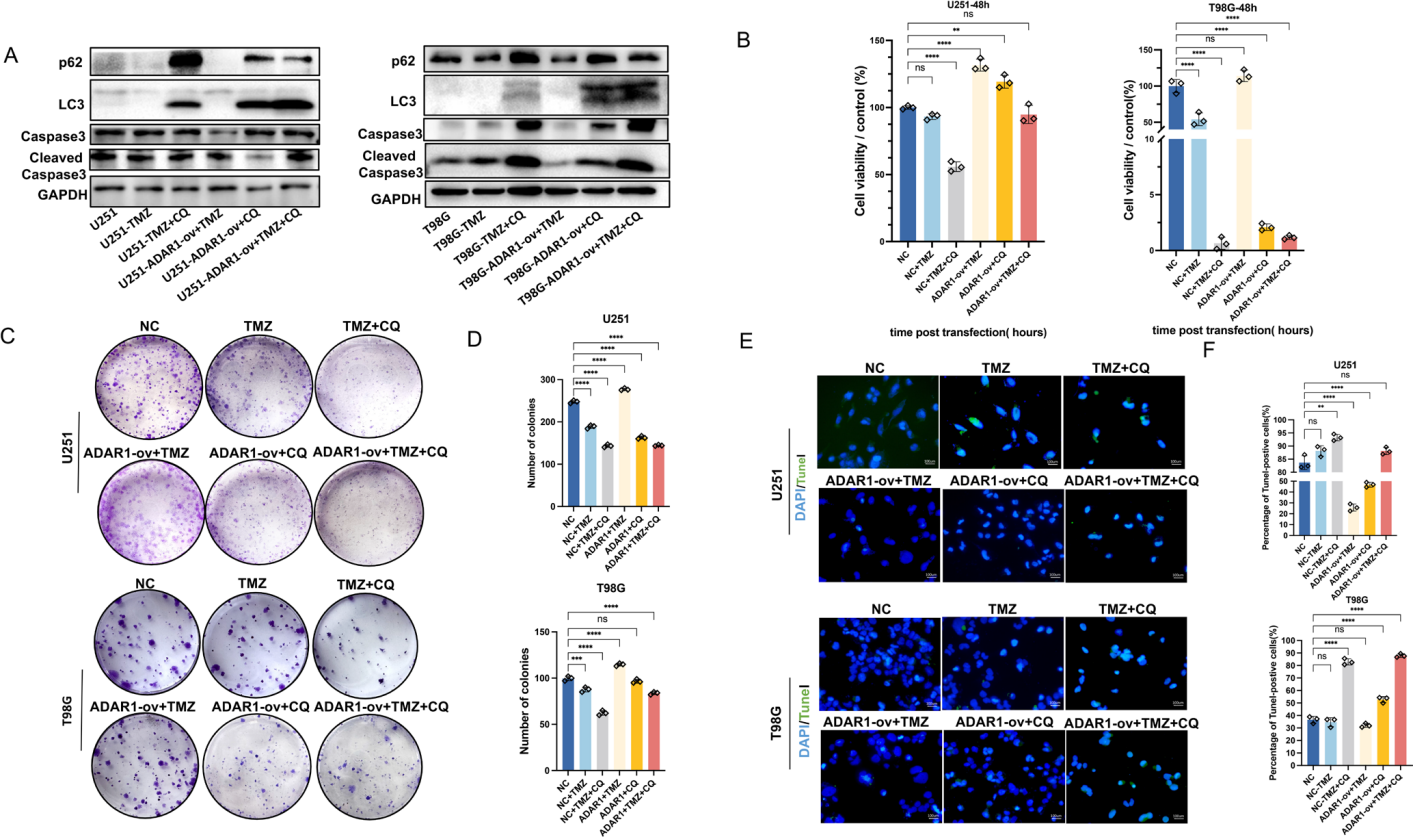
ADAR1 promotes TMZ resistance through autophagy. A, Expression levels of p62, LC3, Caspase3, and Cleaved Caspase3 detected by Western blot in U251 and T98G cells with ADAR1 overexpression or knockdown treated with TMZ and/or 100 μM CQ. B, CCK‐8 assays in U251 and T98G cells with ADAR1 overexpression or knockdown treated with 100 μM TMZ, for 24 h and/or 100 μM CQ, for 17 h. Statistical stest. C, D, Colony formation assay was performed to evaluate the viability of U251 and T98G cells transfected with ADAR1 overexpression or knockdown treated with TMZ, for 24 h and/or 100 μM CQ, for 17 h. Statistical significance was determined by ANOVA. E, F, IF assays showing TUNEL levels (TUNEL in green and nucleus in blue) of U251and T98G cells with ADAR1 overexpression or knockdown treated with TMZ, for 24 h and/or 100 μM CQ, for 17 h. Scale: 100 μm. Statistical significance was determined by ANOVA. Data are shown as mean ± SEM from three independent experiments. Student's *t* test. *p < 0.05, **p < 0.01, ****p < 0.0001.

Autophagy is a lysosomal degradation pathway that supports the clearance of protein aggregates and damaged subcellular structures to maintain homeostasis [25]. In addition, the downregulation of ADAR1 can be restored through autophagy inhibition [26]. Extensive research has shown that manipulating autophagy in glioma treatment typically has two effects. One of these strategies involves overcoming the protective effects of autophagy induced by drugs and reusing lethal conditions to induce cell death. For example, clinically available doses of TMZ have been used to induce autophagy in glioma cells to survive under adverse conditions [27]. To verify whether ADAR1 can enhance resistance to TMZ through autophagy, glioma cell lines (U251 and T98G) overexpressing ADAR1 were treated with TMZ, and CQ was used to suppress autophagy. Western blotting showed that overexpression of ADAR1 inhibited TMZ‐induced cell apoptosis, while the autophagy inhibitor CQ restored this inhibitory effect, as evidenced by the detection of induced Caspase3 in U251 and T98G cells (Figure 4A). To validate this conclusion, we treated the ADAR1‐overexpressing glioma cell line U251 with TMZ and another autophagy inhibitor (bafilomycin). The Western blotting results are consistent with the previous results (Figure S5A). The CCK8 assay results also showed that when ADAR1 was overexpressed, the resistance of the glioma cell lines U251 and T98G to TMZ increased, resulting in increased cell viability. However, the addition of the autophagy inhibitor CQ weakened this resistance to some extent (Figure 4B). Plate cloning experiments also showed that autophagy inhibitors can also inhibit the promotive effect of ADAR1 overexpression on cell proliferation, further indicating that ADAR1 can promote drug resistance to TMZ through the autophagy pathway (Figure 4C,D). We also verified the effect of inhibiting autophagy on cell apoptosis through reverse immunofluorescence, and the results showed that cells overexpressing ADAR1 had a decrease in apoptosis compared to that of the control group after a combined action with TMZ, while adding CQ significantly increased apoptosis (Figure 4E,F). These results indicate that ADAR1 promotes resistance to TMZ through autophagy.

### 3.5 ADAR1 Regulates Selective Autophagy Through p62

**FIGURE 5:**
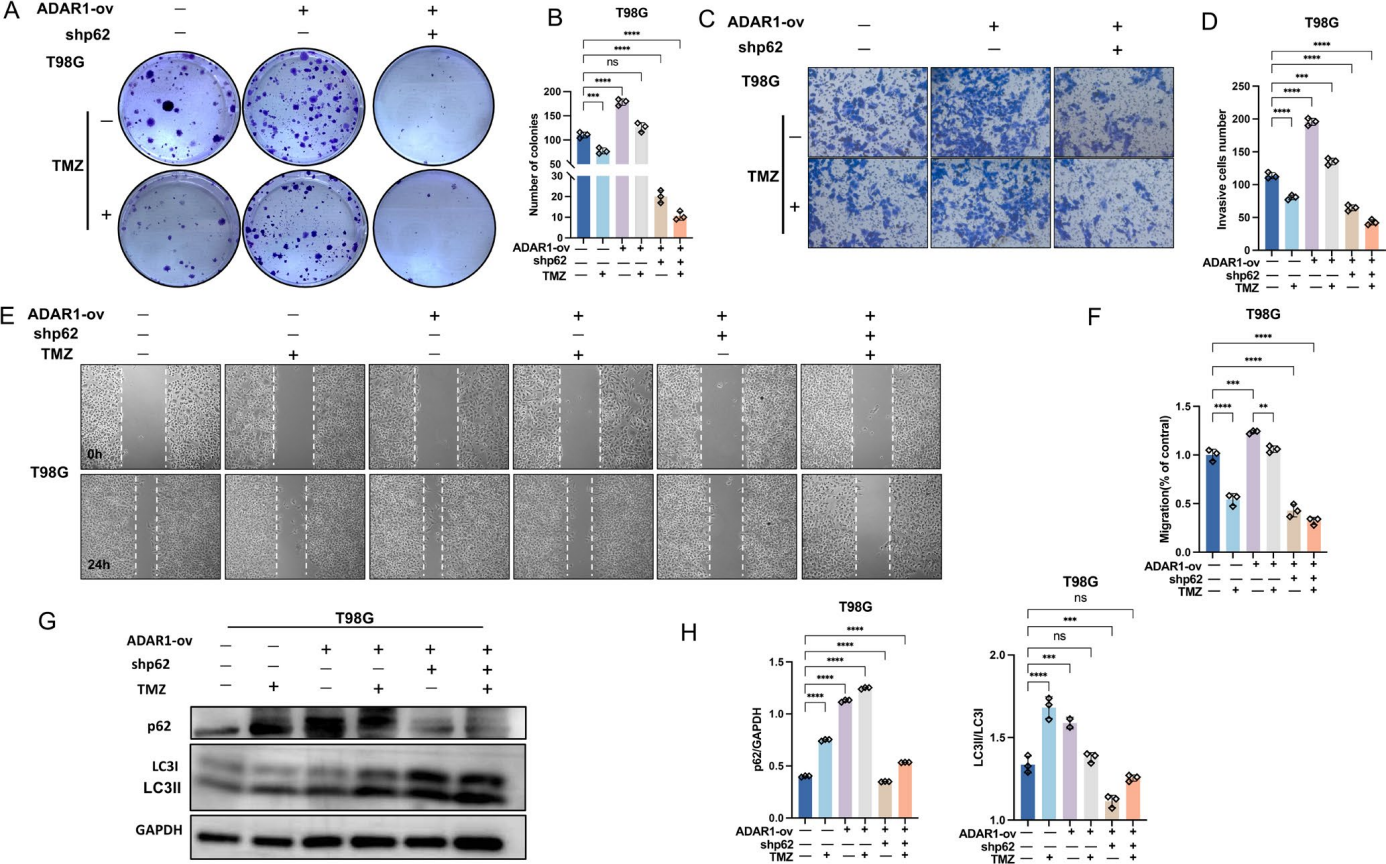
ADAR1 regulates selective autophagy through P62 regulation. A, B, Colony formation assay was performed to evaluate the viability of T98G cells treated with TMZ. Statistical significance was determined by ANOVA. C, D, Wound healing migration assays were performed in T98G cells treated with TMZ for 24 h. Statistical significance was determined by ANOVA. E, F, Transwell invasion assays in T98G cells treated with TMZ for 24 h. G, H, Expression levels of p62 and LC3 detected by Western blot in T98G cells treated with TMZ for 24 h. Statistical significance was determined by ANOVA. Data are shown as mean ± SEM from three independent experiments. Student's *t* test. *p < 0.05, **p < 0.01, ***p < 0.001, ****p < 0.0001.

Research has found that autophagy has a high degree of selectivity toward transported goods. Unlike nonselective autophagy, selective autophagy selects specific substrates in a signal‐dependent manner and is mediated by autophagy adaptor proteins, including p62, NBR1, TAX1 binding proteins, etc. [28, 29] Since p62 is an important adaptor protein involved in selective autophagy, we speculate that ADAR1 regulates selective autophagy through p62. To verify our hypothesis, we used shRNA to knock down p62 in glioma cell lines (T98G) with ADAR1 overexpression through adenovirus infection, because in the previous immunofluorescence results, we found that autophagy was most pronounced in the T98G cell line. Subsequently, through cloning, we found that under the treatment of TMZ, the proliferation ability of glioma cells significantly weakened after p62 knockdown and even reversed the phenomenon of ADAR1 overexpression (Figure 5A,B). To evaluate the migration ability of cells in vitro, we subsequently conducted cell monolayer damage experiments and Transwell experiments. The experimental results confirmed that p62 also affects the migration ability of cells in vitro (Figure 5C–F). By analyzing the changes in cell damage area and the ability of cells to pass through the upper layer of the small chamber, it is not difficult to see that knockdown of p62 inhibits cell migration. This also preliminarily confirms our hypothesis that regulates selective autophagy through p62 regulation. In order to observe the effect of knocking down p62 on autophagy, we also used T98G cells to verify the expression levels of LC3 and p62 through Western blotting. We found that the expression level of p62 protein in two cells significantly decreased after knocking down p62, which proves the effectiveness of our knocking down. After p62 silencing, the phenomenon of increased LC3II/LC3I protein after ADAR1 overexpression was reversed (Figure 5G,H). This once again confirms our hypothesis that ADAR1 regulates selective autophagy through p62.

### 3.6 ADAR1 Regulates Selective Autophagy in an Editing‐Dependent Way

**FIGURE 6:**
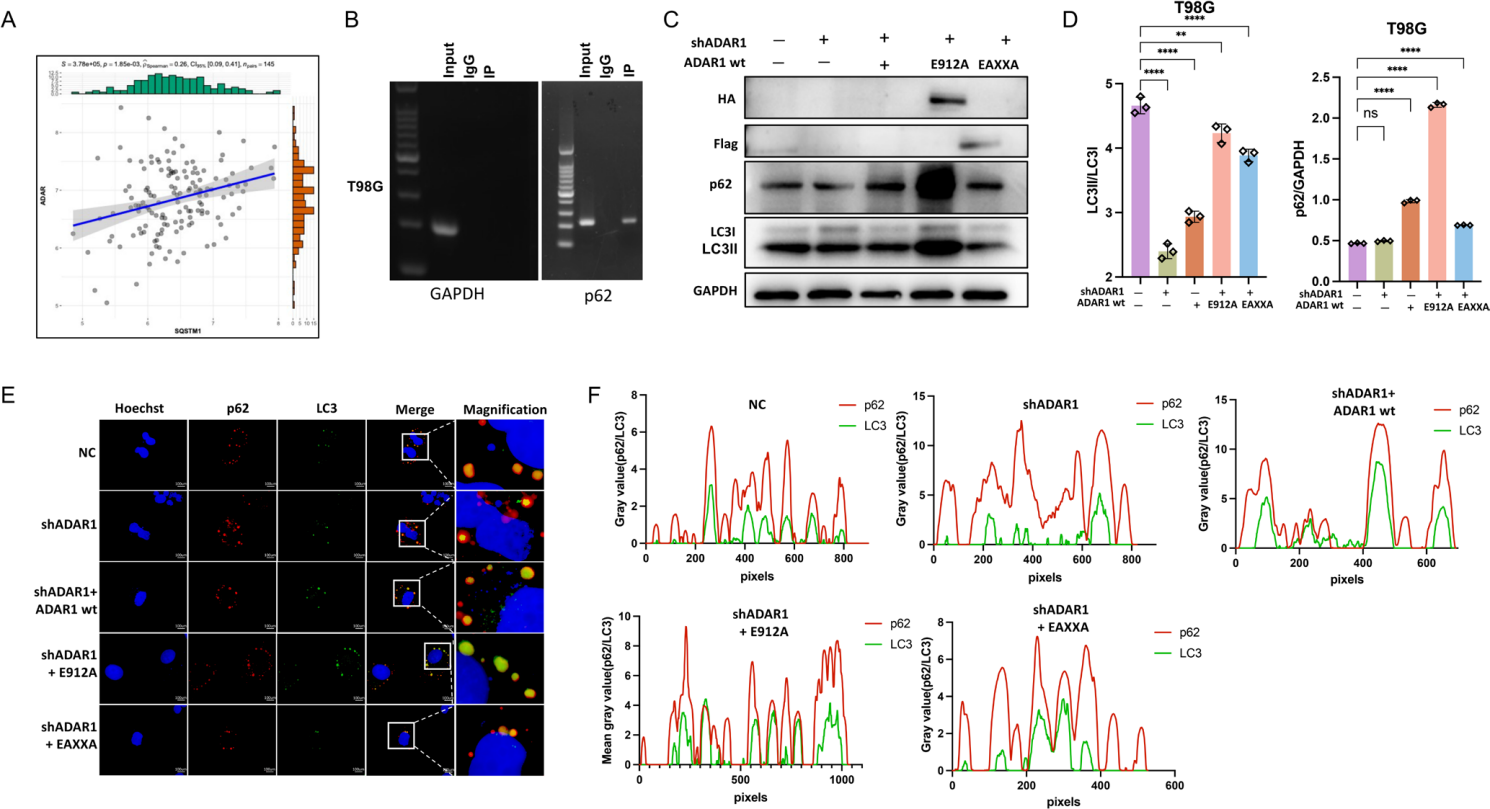
ADAR1 regulates selective autophagy in an editing‐dependent way. A, Correlation between ADAR1 and p62 (SQSTM1) in TCGA database. Statistical significance was determined by Wilcoxon's signed‐rank test (*p* = 1.85e−0.3). B, ADAR1 RNA immunoprecipitation (RIP) to pull down p62 mRNA in indicated T98G. GAPDH served as nonspecific control. C, D, Expression levels of HA‐Tag, Flag‐Tag, p62, and LC3 detected by Western blot in T98G cells, Statistical significance was determined by ANOVA. E, Immunofluorescence images of p62 and LC3 showed that p62 (red) is co‐localized with LC3 (green). The scale bar in the column represents 100 μm. F, Co‐localization analysis of p62 and LC3. Data are shown as mean ± SEM from three independent experiments. Student's *t* test. **p < 0.01, ***p < 0.001, ****p < 0.0001.

Although we have validated that ADAR1 regulates selective autophagy through p62, we still do not know ADAR1 regulates autophagy in what form. However, A‐to‐I RNA editing catalyzed by ADARs (adenosine deaminases acting on RNA) is the most common RNA editing event in mammals, with over 85% of RNA potentially being edited in the coding and/or noncoding regions [30, 31]. By analyzing GBM data in the TCGA database, we found a positive correlation between ADAR1 and p62 (Figure 6A). Subsequently, we also found that ADAR1 binds to p62 in T98G cells by ADAR1 RNA immunoprecipitation PCR (RIP‐PCR) (Figure 6B). However, we still do not know how ADAR1 regulates autophagy; in order to further investigate this issue, we infected T98G cells which already knocked down ADAR1 with a lentivirus consisting of wild‐type (WT) ADAR1 (p110 isomer), a mutant variant of dsRNA binding (EAXXA [32] with Flag‐Tag), and a mutant (E912A [33] with HA‐Tag) containing a point mutation at its catalytic site. Through the Western blotting experiment, we found that both LC3 protein and p62 protein were expressed at the same level in ADAR1 WT and ADAR1 EAXXA, but LC3II/LC3I and p62 were significantly increased in ADAR1 E912A (Figure 6C,D). Our analysis suggests that this phenomenon is due to the accumulation of these two proteins after autophagy is inhibited in ADAR1 E912A. This preliminarily confirms that ADAR1 regulates selective autophagy through the editing form. Immunofluorescence staining showed co‐localization of two specific markers of autophagy (LC3 and p62) (Figure 6E). Subsequent quantitative analysis also confirms that this phenomenon colocalization is a significant enhancement in ADAR1 E912A (Figure 6F), which is consistent with the protein results. Therefore, we speculate that ADAR1 regulates selective autophagy in an editing‐dependent way.

### 3.7 ADAR1 Promotes TMZ Resistance in an Orthotopic GBM Model In Vivo

**FIGURE 7:**
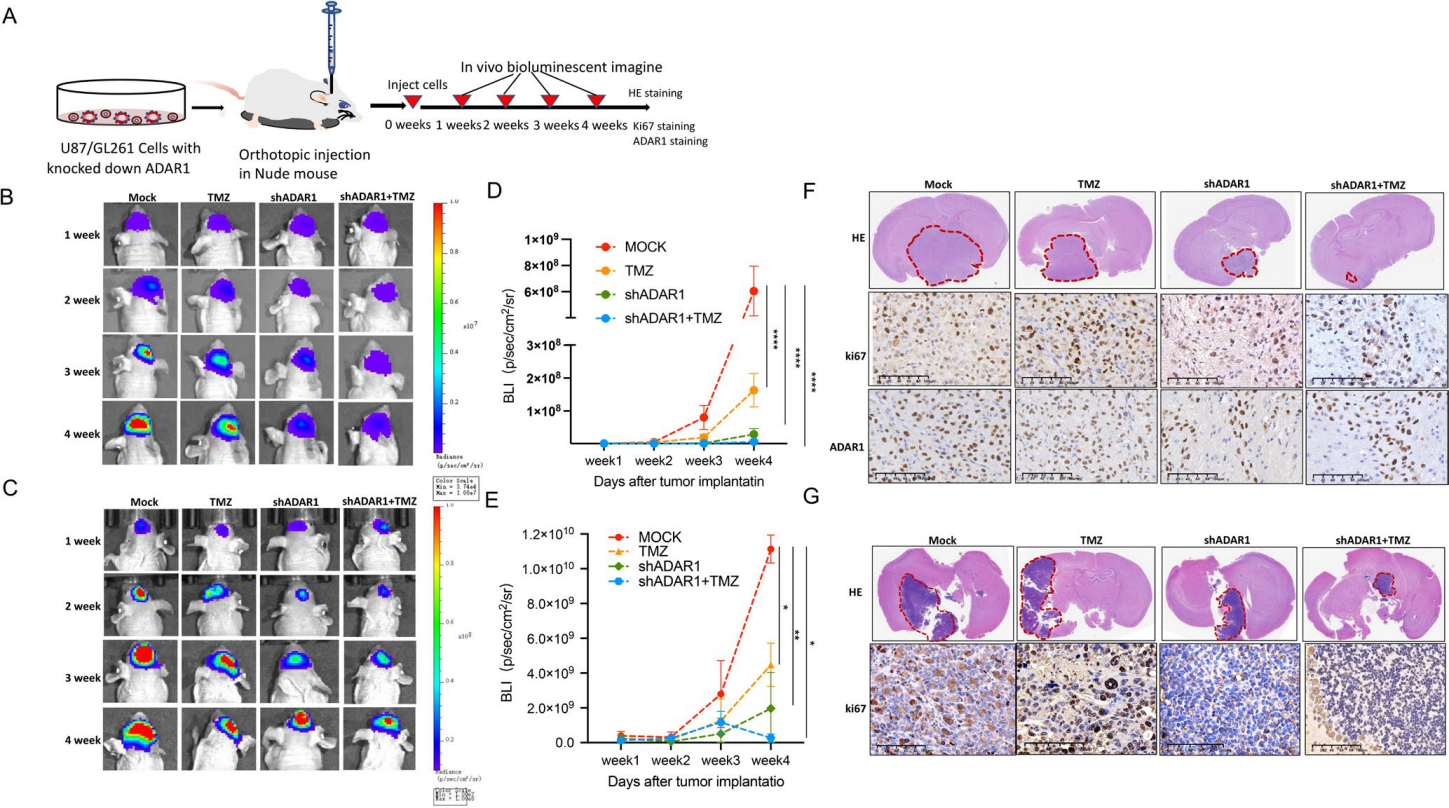
ADAR1 promotes TMZ resistance in an orthotopic GBM model in vivo. A, Experimental design for the role of ADAR1 in TMZ resistance in glioma. B, Tumor formation was detected using bioluminescence imaging (BLI) on weeks 1, 2, 3 and 4 after tumor implantation (U87). C, Tumor formation was detected using bioluminescence imaging (BLI) on weeks 1, 2, 3 and 4 after tumor implantation (GL261). D, BLI analysis was performed to evaluate tumor growth on weeks 1, 2, 3 and 4, Statistical significance was determined by 2‐way ANOVA with Dunnett's multiple‐comparison test (U87). E, BLI analysis was performed to evaluate tumor growth on weeks 1, 2, 3 and 4, Statistical significance was determined by 2‐way ANOVA with Dunnett's multiple‐comparison test (GL261). F, Representative images of H&E staining and immunohistochemical staining of Ki67 and ADAR1 in mouse tissues(U87). G, Representative images of H&E staining and immunohistochemical staining of Ki67 in mouse tissues (GL261). Data are shown as the means ± SEMs from three independent experiments. Student's *t* test. *p < 0.05, **p < 0.01, ****p < 0.0001.

To further confirm the effect of ADAR1‐mediated TMZ resistance on glioma cells in vivo, we established an orthotopic glioma model using U87 and GL261 stable cell lines infected with lentiviruses expressing luciferase and carrying ADAR1 shRNA or TMZ (Figure 7A). After glioma implantation, bioluminescence imaging analysis of the nude mice showed that the shADAR1 and TMZ treatment groups exhibited significantly smaller intracranial tumour volumes in nude mice than the shADAR1 or TMZ alone groups (Figure 7B–E). H&E and immunohistochemical staining of the brain tissues from orthotopic GBM model mice showed that the tumour volume in the shADAR1 groups and TMZ groups was significantly lower than that in the shADAR1 and/or TMZ group (Figure 7F,G). These findings suggest that ADAR1 promotes TMZ resistance in glioma. In summary, our work has preliminarily elucidated the impact of ADAR1 on the progression of glioma and the mechanism of TMZ resistance. These data indicate that ADAR1 mediated RNA editing can regulate selective autophagy through p62, endowing it with chemotherapy resistance and allowing it to survive under chemotherapy induced stress. In terms of treatment, adding ADAR1 inhibitors to the chemotherapy regimen may improve the therapeutic effect.

## 4 Discussion

Most glioblastomas are incurable and have poor clinical prognoses. Although TMZ is a commonly used chemical drug for the treatment of glioblastoma, its drug resistance is a concern due to the lack of fully replaceable treatment methods for glioblastoma [34]. Therefore, a systematic understanding of the mechanisms related to TMZ resistance is crucial for improving the antitumor effect of TMZ in glioma patients. Our study showed that the expression of ADAR1 is positively correlated with GBM grade and that high ADAR1 expression is significantly correlated with TMZ resistance. From a mechanistic perspective, we elucidated that after treatment with TMZ, elevated ADAR1 promotes resistance to TMZ through autophagy.

ADAR1 is encoded by the ADAR gene, is located at the chromosomal region 1q21, and is ubiquitously expressed in mammals [35]. The development of cancer is increasingly recognized as a heterogeneous clonal population that follows Mendelian characteristics [36]. Our bioinformatics analysis of the CGGA database showed that the expression of ADAR1 was greater in human glioma tissues than in normal tissues, and increased expression of ADAR1 was associated with poor prognosis in glioma patients. We also found a significant correlation between the expression of ADAR1 and the grading of human glioma according to the World Health Organization. Knocking out ADAR1 can significantly inhibit tumor growth both in vivo and in vitro. ADAR1 knockout significantly enhanced the antitumor activity of TMZ in glioma cells, indicating that ADAR1 is involved in the resistance of glioma cells to TMZ. In addition, ADAR1 may regulate TMZ resistance through autophagy.

Autophagy is a closely coordinated process in which misfolded proteins, damaged or aged organelles, and mutated proteins are isolated in double‐membrane vesicles called autophagosomes, which then fuse into lysosomes, ultimately leading to the degradation of isolated components [37]. Autophagy plays a dual role in cancer, inhibiting the growth of benign tumors while promoting the growth of advanced cancers. In the past decade, many research groups have identified autophagy as a potential therapeutic target for cancer treatment. Increasing evidence support the role of autophagy in promoting established tumor resistance in cancer treatment. This was first confirmed in a therapeutic mouse model of lymphoma, in which the inhibition of autophagy significantly enhanced the efficacy of chemical drugs [38]. Our research results not only demonstrate the relationship between ADAR1 and glioma but also provide evidence that autophagy is an influencing factor in the promotion of TMZ resistance by ADAR1.

In mammals, RNA editing alters the transcriptional sequence of expressed RNA without affecting the DNA sequence [39, 40]. A‐to‐I RNA editing catalyzed by ADARs (adenosine deaminases acting on RNA) is the most common RNA editing event in mammals, with over 85% of RNA possibly edited in coding and/or noncoding regions [41, 42]. To investigate the regulatory mechanism of ADAR1, we infected glioma cells with a lentivirus consisting of wild‐type (WT) ADAR1 (p110 isomer), a mutant variant of dsRNA binding (EAXXA), and a mutant (E912A) containing a point mutation at its catalytic site. We found that autophagy was inhibited by ADAR1 E912A but not ADAR1 WT or ADAR1 EAXXA. Therefore, we speculate that ADAR1 regulates autophagy through p62 and is in an editing‐dependent way.

## 5 Conclusions

In summary, our research results revealed the relationship between ADAR1 levels and the response of glioma patients to TMZ treatment. In addition, the expression of ADAR1 is related to survival time and prognosis, and this protein can serve as a biomarker for predicting the prognosis of glioma patients. In addition, ADAR1 enhances the resistance of glioma to TMZ in glioma through autophagy, revealing a new approach for treating glioma. Mechanistically, we speculate that ADAR1 is selectively autophagy‐mediated by p62 and is regulated in an editing‐dependent way. However, there is still a lack of small‐molecule compounds targeting ADAR1, which will be the direction of future efforts. Therefore, inhibiting ADAR1 may be a promising treatment strategy for enhancing the sensitivity of glioma to TMZ treatment.

## Author Contributions

J.W., L.M., and X.L. were responsible for the study's design. Y.Z., H.G., and J.B. authored the manuscript and conducted most of the experiments. W.W., L.W., Z.L., and Y.Q. performed the data analysis and prepared the figures, while Q.W. and L.Z. meticulously revised the manuscript. All authors meticulously reviewed and approved the final manuscript.

## Conflicts of Interest

The authors declare no conflicts of interest.

## Ethics Statement and Consent

Ethics approval and consent to participate all experiments were performed in accordance with the standards of the ethics committee of the First Affiliated Hospital of Zhengzhou University.

## Supporting information


**Figure S1.** ADAR1 is correlated with patient prognosis and promotes TMZ resistance in glioma cells. (A) Expression level of ADAR1 mRNA in GBM and LGG tissues compared to normal tissues in GEPIA databases. Statistical significance was determined by Wilcoxon’s signed‐rank test. (B) Kaplan–Meier analysis of ADAR1 expression in GBM and LGG in the CGGA database. *p* value was determined by log‐rank test, *p* = 0.017. (C) Expression levels of Caspase3, Cleaved Caspase3, Bax, Bcl‐2 detected by Western blot in U87 cells with ADAR1 overexpression or knockdown treated with TMZ for 24 h.
**Figure S2.** Verifying the effect of ADAR1 knockdown and overexpression. (A‐C) RT–qPCR analysis was performed to detect the expression of ADAR1 in U251, T98G and U87 cells transfected with ADAR1 overexpression or knockdown. Statistical significance was determined by ANOVA. (D) Expression levels of ADAR1 detected by Western blot in U251, T98G and U87 cells with ADAR1 overexpression or knockdown. (E) IF assays showing ki67 levels (ki67 in green and nucleus in blue) of U251 cells with ADAR1 overexpression or knockdown treated with 100 μM TMZ. Scale: 100 μm. The statistics of IF assays are shown in bar plots. Data are shown as mean ± SEM from three independent experiments. Student’s *t* test. **p < 0.01, ****p < 0.0001.
**Figure S3.** Verifying the effect of ADAR1 on the migration ability of U87 cells. (A) Wound healing migration assays were performed inU87 cells transfected with ADAR1 overexpression or knockdown treated with TMZ for 24 h. Statistical significance was determined by ANOVA. (B) Transwell invasion assays in U87 cells transfected with ADAR1 overexpression or knockdown treated with TMZ for 24 h. Statistical significance was determined by ANOVA. Data are shown as mean ± SEM from three independent experiments. Student’s *t* test. **p < 0.01, ***p < 0.001, ****p < 0.0001.
**Figure S4.** ADAR1 promotes TMZ to induce the activation of autophagy in U87 cells. (A) Expression levels of p62 and LC3 detected by Western blot inU87 cells with ADAR1 overexpression or knockdown treated with TMZ for 24 h. (B) Immunofluorescence images of p62 and LC3 showed that p62 (red) is co‐localized with LC3 (green). The scale bar in the column represents 100 μm. The statistics of IF assays are shown in bar plots, Statistical significance was determined by ANOVA. Statistical significance was determined by ANOVA. Data are shown as mean ± SEM from three independent experiments. Student’s *t* test. *p < 0.05, ***p < 0.001, ****p < 0.0001.
**Figure S5.** ADAR1 promotes TMZ resistance through autophagy. A, Expression levels of p62, LC3, Caspase3, Cleaved Caspase3 detected by Western blot in U251, and T98G cells with ADAR1 overexpression or knockdown treated with TMZ, for 24 h and/or 100 nM Bafilomycin, for 17 h.

## Data Availability

These data and materials supporting the conclusion of this manuscript are included in the article.
