# Supplementary material for: ADAR1 Promotes the Progression and Temozolomide Resistance of Glioma Through p62‐Mediated Selective Autophagy
Source: CNS Neurosci Ther. 2025 Jan 18;31(1):e70168. doi: 10.1111/cns.70168 (PMC11742087; doi:10.1111/cns.70168)

Supplementary Fig.S1 ADAR1 is correlated with patient prognosis and promotes TMZ resistance in Glioma cells

A

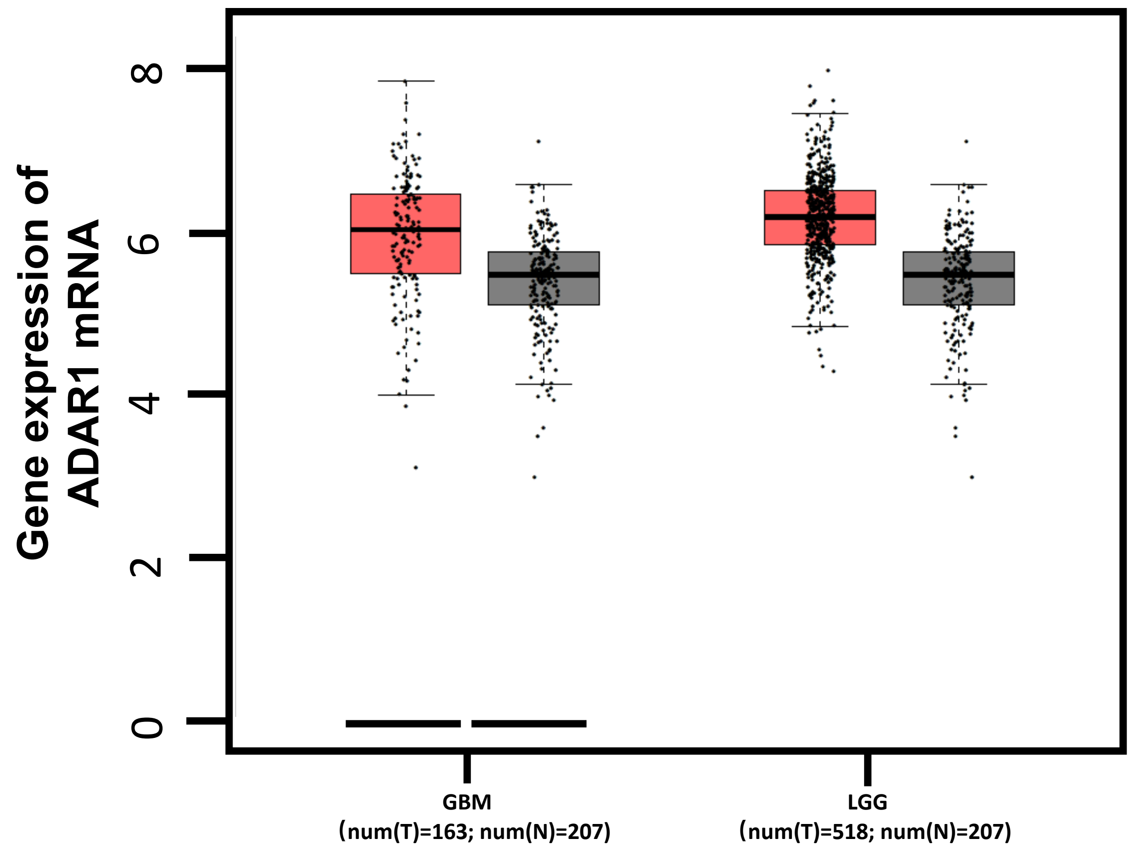

B

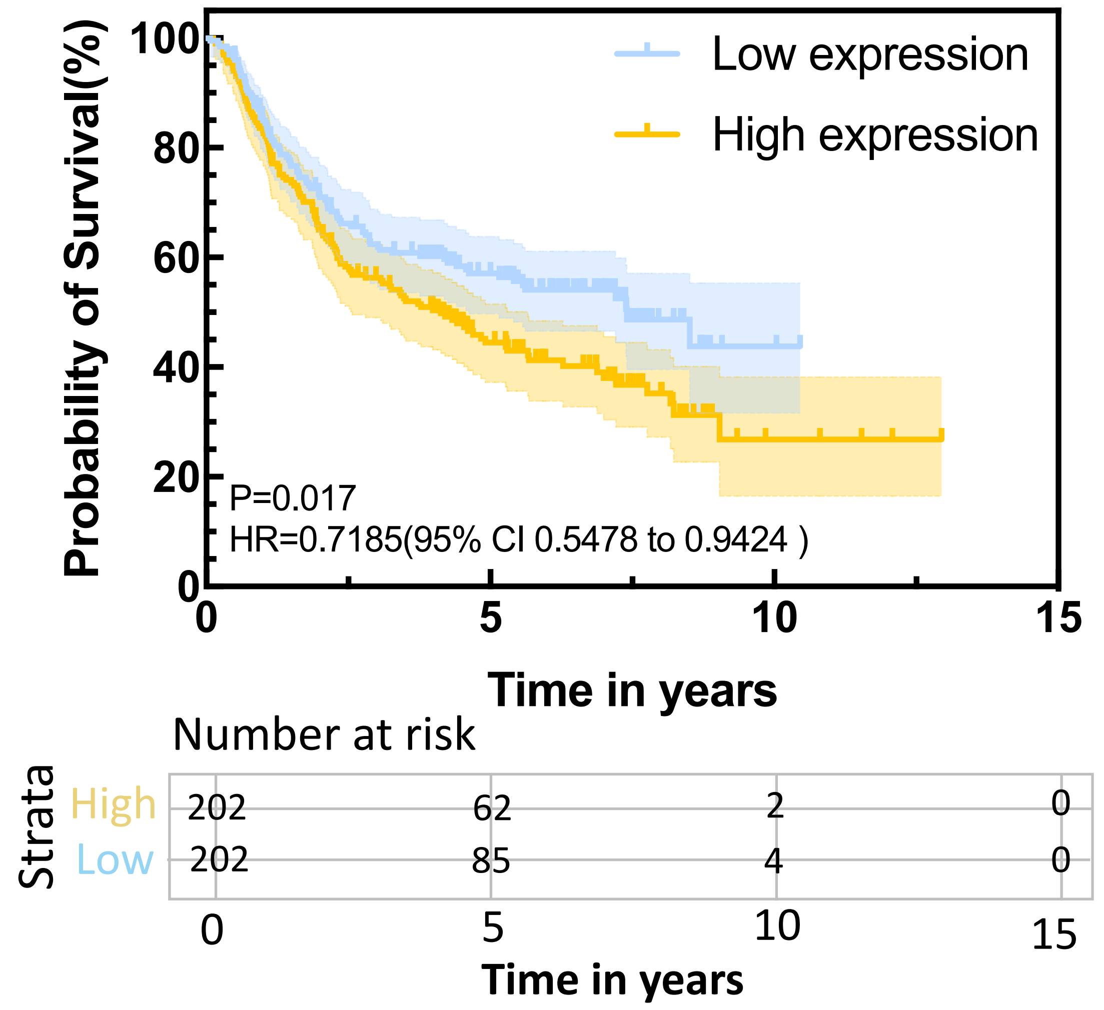

C

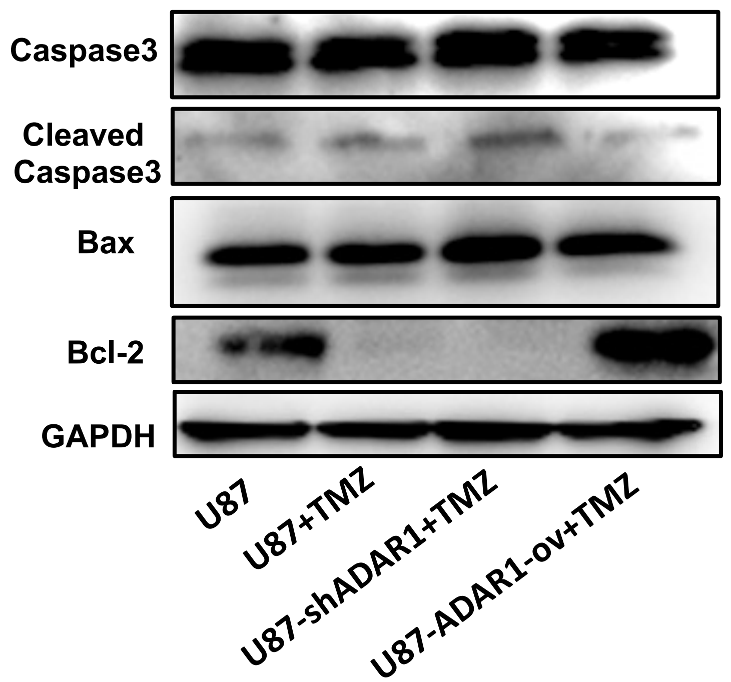

Supplementary Fig.S2 Verify the effect of ADAR1 knockdown and overexpression

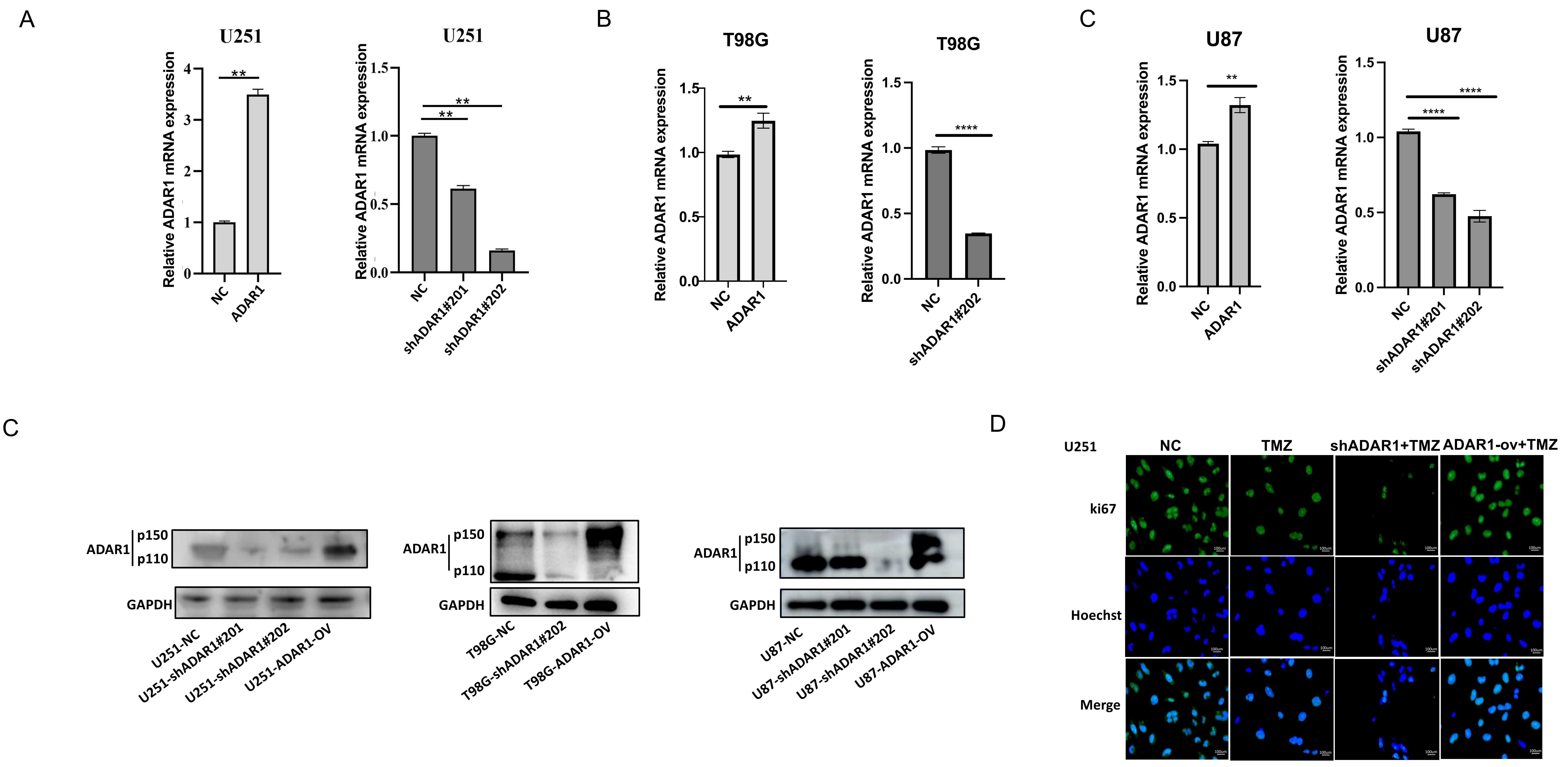

Supplementary Fig.S3Verify the effect of ADAR1 on the migration ability of U87 cells

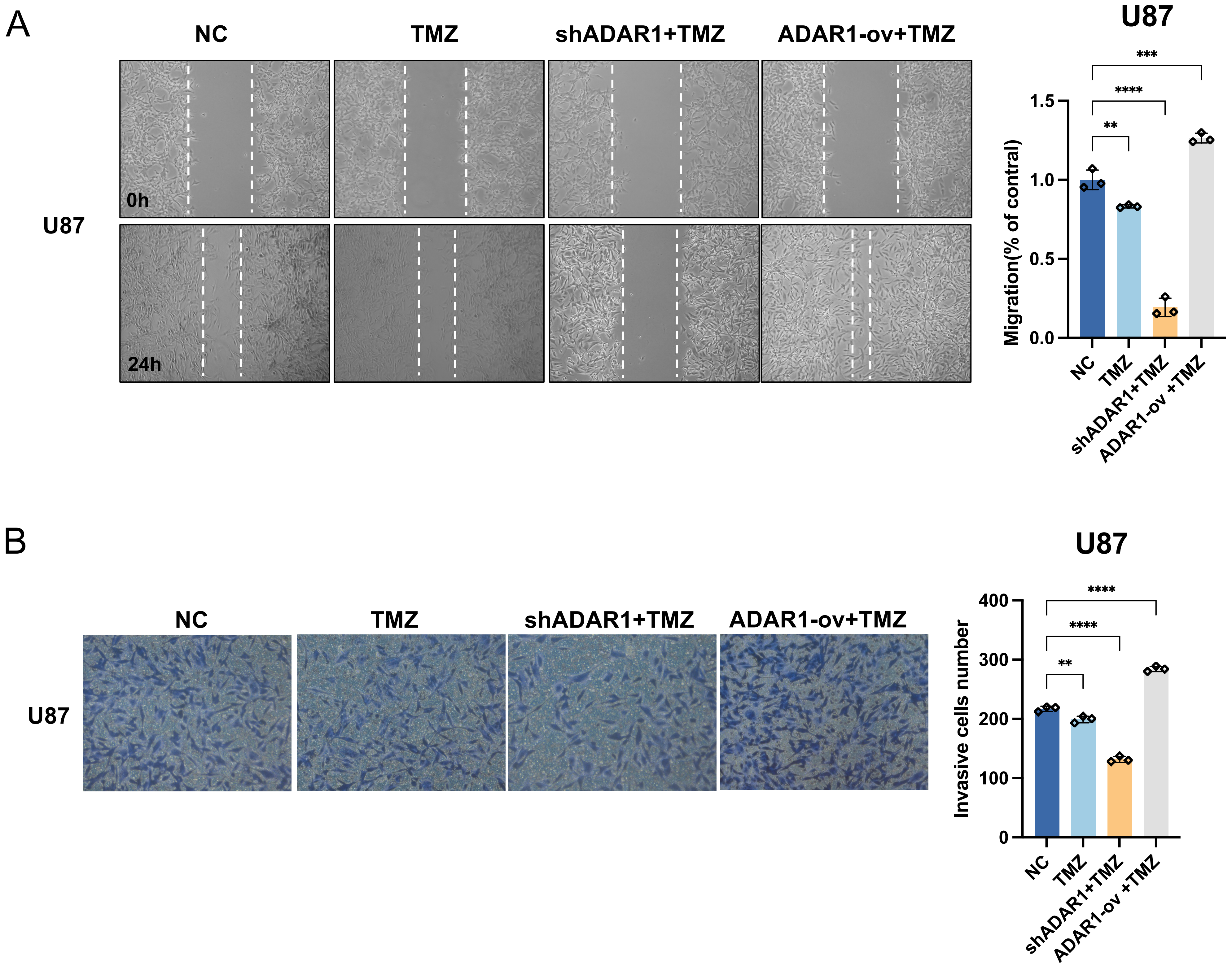

Supplementary Fig.S4 ADAR1 promotes TMZ to induce the activation of autophagy in U87 cells

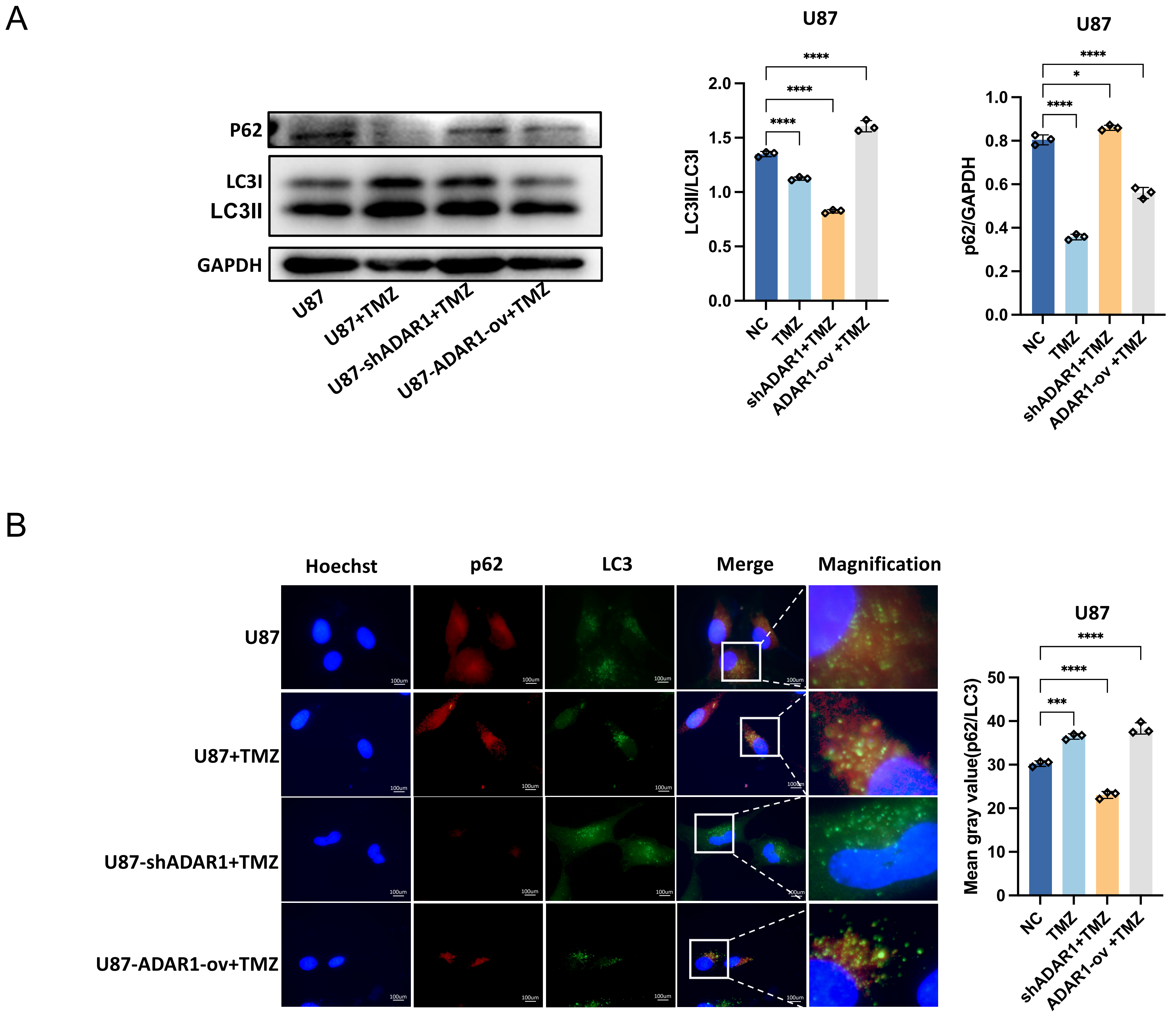

Supplementary Fig.S5 ADAR1 promotes TMZ resistance through autophagy.

A

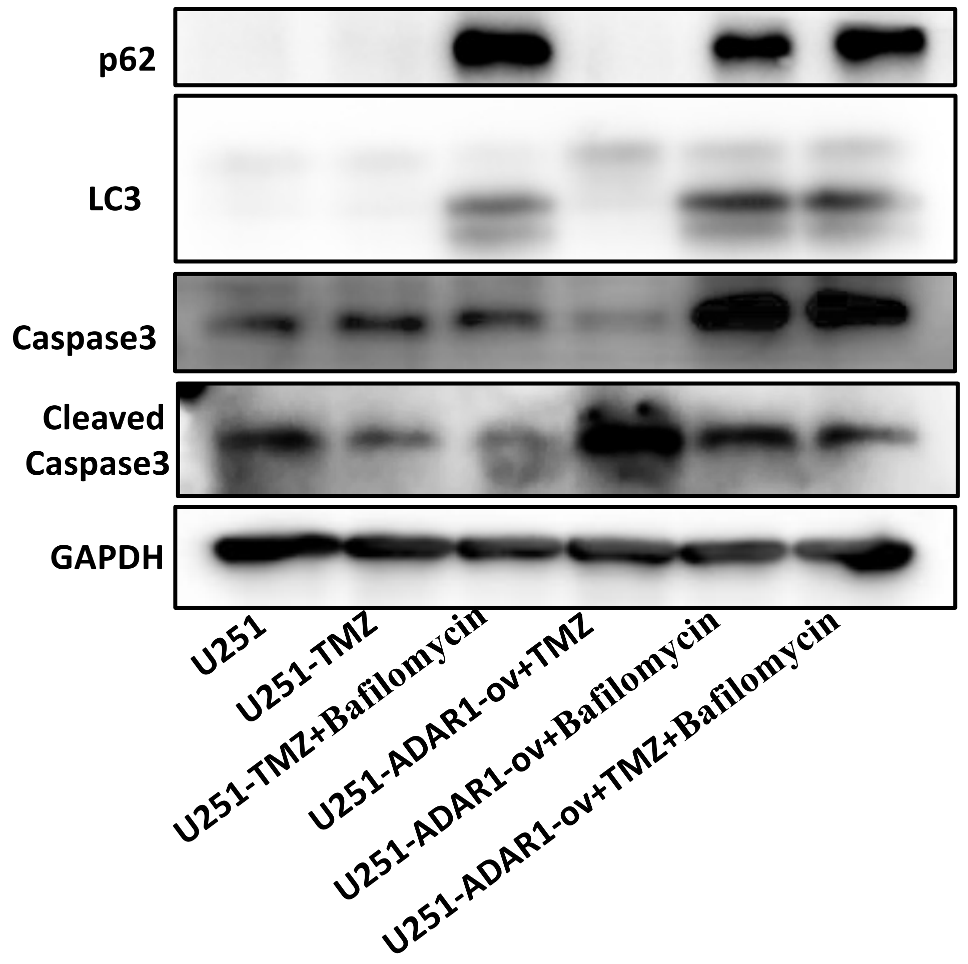

Supplement: Supplementary file 1 — Figure S1. ADAR1 is correlated with patient prognosis and promotes TMZ resistance in glioma cells. (A) Expression level of ADAR1 mRNA in GBM and LGG tissues compared to normal tissues in GEPIA databases. Statistical significance was determined by Wilcoxon’s signed‐rank test. (B) Kaplan–Meier analysis of ADAR1 expression in GBM and LGG in the CGGA database. p value was determined by log‐rank test, p = 0.017. (C) Expression levels of Caspase3, Cleaved Caspase3, Bax, Bcl‐2 detected by Western blot in U87 cells with ADAR1 overexpression or knockdown treated with TMZ for 24 h. Figure S2. Verifying the effect of ADAR1 knockdown and overexpression. (A‐C) RT–qPCR analysis was performed to detect the expression of ADAR1 in U251, T98G and U87 cells transfected with ADAR1 overexpression or knockdown. Statistical significance was determined by ANOVA. (D) Expression levels of ADAR1 detected by Western blot in U251, T98G and U87 cells with ADAR1 overexpression or knockdown. (E) IF assays showing ki67 levels (ki67 in green and nucleus in blue) of U251 cells with ADAR1 overexpression or knockdown treated with 100 μM TMZ. Scale: 100 μm. The statistics of IF assays are shown in bar plots. Data are shown as mean ± SEM from three independent experiments. Student’s t test. **p < 0.01, ****p < 0.0001. Figure S3. Verifying the effect of ADAR1 on the migration ability of U87 cells. (A) Wound healing migration assays were performed inU87 cells transfected with ADAR1 overexpression or knockdown treated with TMZ for 24 h. Statistical significance was determined by ANOVA. (B) Transwell invasion assays in U87 cells transfected with ADAR1 overexpression or knockdown treated with TMZ for 24 h. Statistical significance was determined by ANOVA. Data are shown as mean ± SEM from three independent experiments. Student’s t test. **p < 0.01, ***p < 0.001, ****p < 0.0001. Figure S4. ADAR1 promotes TMZ to induce the activation of autophagy in U87 cells. (A) Expression levels of p62 and LC3 detected by We [file CNS-31-e70168-s001.pdf]
